# How Alexithymia Increases Mental Health Symptoms in Adolescence: Longitudinal Evidence for the Mediating Role of Emotion Regulation

**DOI:** 10.1007/s42761-025-00318-9

**Published:** 2025-07-08

**Authors:** Jack D. Brett, Majid Zarei, David A. Preece, Rodrigo Becerra, James J. Gross, Mahdi Mazidi

**Affiliations:** 1 The University of Western Australia, 35 Stirling Hwy, Crawley, WA 6009 Australia; 2 Telethon Kids Institute, Northern Entrance, Perth Children’s Hospital, 15 Hospital Ave, Nedlands, WA 6009 Australia; 3 University of Social Welfare and Rehabilitation Sciences, Evin, Iran; 4 Curtin University, Kent St, Bentley, WA 6102 Australia; 5 Standford University, 450 Jane Stanford Way, Stanford, CA 94305 USA

**Keywords:** Alexithymia, Psychopathology, Emotion regulation, Longitudinal design

## Abstract

Alexithymia is characterised by difficulties identifying and describing feelings, as well as a lack of focus on feelings. Alexithymia is a transdiagnostic risk factor for developing a wide array of psychopathologies, such as anxiety and depression, with a key hypothesised mechanism being the impairing impact of alexithymia on emotion regulation competency. However, no study has tested whether difficulties with emotion regulation mediate the link between alexithymia and psychopathological symptoms using longitudinal designs. The present study aimed to address this limitation by collecting data from 164 Iranian high school students (73% male; aged 13–18 years old) at two time points, 7 months apart. The results revealed that baseline alexithymia levels not only related to future emotion regulation difficulties but predicted increased emotion regulation difficulties in the future. Furthermore, these increased difficulties in emotion regulation mediated the relationship between baseline alexithymia and worsening of psychological distress (i.e., depression, anxiety, and stress symptoms) over time. This study helps link alexithymia and emotion regulation difficulties and highlights the critical role that alexithymia plays in emotional health and illness during adolescence.

**Supplementary Information:**

The online version contains supplementary material available at 10.1007/s42761-025-00318-9.

Alexithymia is a trait characterised by difficulties identifying one’s feelings (DIF), difficulties describing one’s feelings (DDF), and an externally oriented thinking style (EOT) involving a tendency not to focus attention on one’s feelings (Luminet & Nielson, 2024; Preece & Gross, 2023). Since the term was first coined in the 1970s (Sifneos, 1973), alexithymia has been touted as a key transdiagnostic risk factor for developing psychopathologies, such as anxiety and depressive disorders (e.g., Taylor et al., 1999). Levels of alexithymia vary in the general population, and individuals with greater levels of alexithymia generally report poorer emotional wellbeing, including increased mental health symptoms (Preece et al., 2024), depression (Li, Stuart et al., 2015; Li, Zhang et al., 2015), anxiety (De Berardis et al., 2008), eating disorder symptoms (Muir et al., 2024), substance use (Honkalampi et al., 2022), self-harm (Norman et al., 2020), and suicidal ideation and behavior (Hemming et al., 2019).

One life stage of particular research interest for the development of mental health symptoms is adolescence (Mclaughlin et al., 2015). Adolescents face several significant stressors (e.g., academic pressures and increased importance of peer relationships) and are vulnerable to developing a range of psychopathologies (Lynch et al., 2021), which often persist into adulthood, leading to chronic and severe impacts (Johnson et al., 2018). Runcan’s (2020) review identified that alexithymia in adolescents has been linked to myriad emotional and behavioral challenges, including depression, anxiety, and stress.

Why does alexithymia seem to put some adolescents at greater risk for mental health problems? A potential explanation regards alexithymia’s impact on effective emotion regulation. Emotion regulation occurs when an individual attempts to influence their own emotion trajectory (Gross, 2015). Young et al.’s (2019) review noted that, on average, during adolescence, as compared to younger developmental stages, individuals being to increase their use of adaptive strategies (e.g., cognitive reappraisal) and reduce maladaptive strategies (e.g., avoidance), which may reduce the likelihood of them experiencing anxiety and depression symptoms (Schäfer et al., 2017). However, there are significant individual differences in adolescents in their emotion regulation development and as research has linked alexithymia with impairing individuals’ emotion regulation competency (see Preece et al., 2022), a vital question, is whether alexithymia hinders adolescents from developing their emotion regulation skills during this period at the same rate as their peers, potentially contributing to ongoing mental health concerns.

One useful theoretical framework for informing testable predictions is the attention-appraisal model of alexithymia (Preece et al., 2017), which specifies how alexithymia impairs key stages in emotion processing. The model is based within the broader affective science framework of the process model of emotion regulation (Gross, 2015). These models view emotion processing as unfolding across *situation-attention-appraisal-response* sequences. An emotional response is a type of *situation* or stimulus that can be the target of evaluation. To process the emotion, people first need to focus *attention* on it (i.e., notice it and perceive its features), then *appraise* it in terms of what it is and what it means for them (i.e., interpreting if it is an emotion, and if so, what type of emotion is it). Based on this appraisal, at the *response* stage, people may start to activate a goal to modify the emotion (i.e., emotion regulation). Within this sequence, alexithymia can be understood as one’s degree of difficulties at the *attention* (EOT) and *appraisal* (DIF and DDF) stages of emotion processing. These stages are all crucially linked; for example, difficulties focusing on the emotion at the attention stage will impact how much (or the quality of) information one has at the later appraisal stage, which is the information upon which one needs to base their interpretation of the emotion. Alexithymic difficulties in these processes appear to reflect issues of ability (e.g., low theoretical knowledge of emotions) and/or avoidance (e.g., avoiding focusing on emotions as a coping response; for a review, see Preece & Sikka, 2024).

Because attention to and the appraisal of emotions is a central determinant of downstream emotion regulation decisions (Gross, 2015), the attention-appraisal model predicts that alexithymia should impair emotion regulation and, in turn, put those high in alexithymia at risk of developing psychopathologies characterised by emotion dysregulation (e.g., depression and anxiety). In the first empirical study investigating whether alexithymia increased psychopathology due to impairing emotion regulation competency, Preece et al. (2022) found that, among adults, greater alexithymia was indeed associated with higher psychological distress in a manner that was mediated by greater emotion regulation difficulty.

While promising, there is a need to examine whether such findings generalise to adolescents. Also, several limitations warrant further research. The Preece et al. (2022) study employed a cross-sectional design, which, although it provided evidence consistent with the idea that alexithymia may lead to psychological distress by increasing difficulties in emotion regulation, does not allow for causal inferences. Additionally, they did not investigate whether this mediation was dependent on the emotional valence (i.e., positive or negative) of the emotions being regulated. Adolescents’ ability to regulate both positive and negative emotions may be a vital protective factor against developing anxiety and depression (Young et al., 2019). While the regulation of negative emotions has traditionally been of larger focus in the field, there has been increasing evidence showing the potential importance of difficulty in regulating positive emotions in psychopathology (e.g., bipolar disorders; see Kurtz et al., 2021). For example, Muir et al. (2024) found that difficulty in regulating positive emotions, not negative emotions, mediated the relationship between alexithymia and eating disorder symptoms. However, to date, no study has employed a longitudinal design to investigate whether difficulties in regulating positive or negative emotions mediate the link between alexithymia and psychological distress in adolescents.

## Present Study

The present study seeks to address this limitation, examining alexithymia in adolescents. We use a longitudinal design, examining the potential mediating role of emotion regulation on the relationship between alexithymia and psychological distress (i.e., a composite of depression, anxiety, and stress symptoms). Alexithymia, emotion regulation, and psychological distress were measured across two time points, approximately seven months apart.

We hypothesised that an individual’s current level of alexithymia not only would be related to their future difficulties in emotion regulation and their future levels of psychological distress but also to changes in emotion (dys)regulation and psychological distress levels over time. Furthermore, we hypothesised that a key pathway linking alexithymia to future increases in psychological distress would be the impairing effect of alexithymia on emotion regulation. As such, we predicted that changes in emotion regulation, for both negative and positive emotions, would mediate the relationship between alexithymia and changes in psychological distress.

## Method

### Transparency and Openness

The current study’s de-identified data, data dictionary, and R scripts are openly available in the Open Science Framework at https://osf.io/jwhna/.

### Participants

Participants included adolescents from three elementary schools in Tehran, Iran between Feb 2021 and Sep 2021. To be included participants had to speak and understand Persian fluently, and be under 18 years old; there were no exclusion criteria. A total of 557 students were assessed at Time 1 (T1), with 242 of the initial sample also completing the Time 2 (T2) assessment. The variation in participation primarily occurred because some students changed schools in the new academic year or were absent when the data was collected. The participants who provided data at T1 and T2 had minimal missing data (less than 0.1%), which was replaced using the multiple imputation method (Austin et al., 2021; Li, Stuart et al., 2015; Li, Zhang et al., 2015). Careless responding was also checked according to current guidelines (Curran, 2016; Ward & Meade, 2023). We excluded participants who responded too quickly, spending less than two seconds on average per question (*n* = 14), or those who had incorrect responses to more than one attention check item in any assessment sessions (*n* = 60). Finally, four participants were excluded due to being multivariate outliers using Mahalanobis distance (Becker & Gather, 1999). The final sample, that completed T1 and T2, comprised 164 students (73.17% male). This sample size provided adequate power (i.e., > 0.80) to detect significant mediation effects when the mediator-outcome relationship is *β* = 0.17. The mean age of the final sample (as measured at the second assessment) was 14.77 years (*SD* = 1.34 years), with an age range of 13 to 18 years. On average, 7.37 months (*SD* = 0.93) had elapsed between the two points of data collection. Participants were given bonus course credits for completing the surveys. Data from T1 was previously used in published studies examining the psychometric properties of the Perth Alexithymia Questionnaire (Mazidi, Azizi et al., 2023) and the Perth Emotion Regulation Competency Inventory (Mazidi, Zarei et al., 2023).

### Materials

#### Perth Alexithymia Questionnaire (PAQ)

The PAQ (Preece et al., 2018b) assesses alexithymia levels using 24 items (e.g., “When I’m feeling bad, I can’t tell whether I’m sad, angry or scared”) rated on a 7-point Likert scale (1 = strongly disagree to 7 = strongly agree). It measures all facets of alexithymia across both negative and positive emotions. Subscale and composite scores, including a total score as an overall marker, can be calculated. Higher scores reflect greater alexithymic traits. The PAQ has shown good validity and reliability in assessing alexithymia in Iranian adolescents (Mazidi, Zarei et al., 2023). The PAQ was selected over the Toronto Alexithymia Scale (TAS-20; Bagby et al., 1994) and the Alexithymia Questionnaire for Children (Rieffe et al., 2006) because the TAS-20 has demonstrated inadequate psychometric properties among Iranian adolescents (Mazidi, Zarei et al., 2023), and the psychometric properties of the Alexithymia Questionnaire for Children have not yet been examined in this population.

#### Perth Emotion Regulation Competency Inventory (PERCI)

The PERCI (Preece et al., 2018a) assesses an individual’s emotion regulation competency, or how well an individual can change the experiential and behavioral manifestations of their emotions. Emotion regulation competency is assessed regarding both negative and positive emotions. Items (e.g., “When I’m feeling bad, I’m powerless to change how I’m feeling”) are rated on a 7-point Likert scale, with higher scores reflecting greater emotion dysregulation (i.e., lower regulation ability). Subscale and composite scores can be derived. In this study, we used composite scores for overall difficulties in regulating negative emotions (N-ER) and positive emotions (P-ER). The PERCI has demonstrated good validity for assessing alexithymia in Iranian adolescents and adults (Mazidi, Zarei et al., 2023).

#### Depression Anxiety and Stress Scale-21 (DASS-21)

The DASS-21 (Lovibond & Lovibond, 1995) assesses psychological distress using a four-point Likert scale, where participants rate items measuring depression, anxiety, and stress symptoms. Research suggests that the total score best represents DASS-21 as a general indicator of psychological distress (e.g., Lee et al., 2019). The scale has demonstrated good psychometric properties in Iranian samples (e.g., Asghari et al., 2008).

### Procedure

The current project was carried out in accordance with the Declaration of Helsinki and was approved by the ethics committee of Babol University of Medical Sciences. Parents provided an informed consent form for their adolescents to participate in the study. Additionally, all adolescents assented to participate, ensuring they had the opportunity to agree or decline involvement and withdraw from the study at any time without providing any justification. Participants were given bonus course credits for completing the survey. At each time point, participants completed the Persian version of the questionnaires via Porsline online survey platform (https://survey.porsline.ir/) and with the following order: demographic questions, PERCI, PAQ, and DASS-21.

### Analytic Strategy

#### Change Scores

To measure change in negative and positive emotion regulation difficulties and psychological distress, standardised residual change scores were calculated. Standardised residual change scores are the residuals from bivariate regressions whereby the outcome of interest at T1 predicts that outcome at T2 (Cronbach & Furby, 1970; Traub, 1967). This is akin to including T1 scores as covariates in a multiple regression. Accordingly, those with greater standardised residuals exhibit an increase in that outcome over time. This method provides a measure of change that reliably controls for variability among individual differences at baseline. Three change scores were computed for negative emotion regulation difficulties, positive emotion regulation difficulties, and psychological distress.

#### Alexithymia’s Influence on Change in Emotion Regulation Difficulties and Psychological Distress

To investigate the influence that alexithymia at T1 had on future changes in valence-specific emotion regulation difficulty and psychological distress, a series of bivariate regression analyses were conducted. Firstly, to replicate previous findings suggesting that higher alexithymia is associated with greater emotion regulation difficulties and higher psychological distress, analyses examined whether alexithymia at T1 significantly predicted positive and negative emotion regulation difficulties and psychological distress at T2. Secondly, to examine our first hypothesis that alexithymia predicts *future changes* in emotion regulation and psychological distress, analyses examined whether alexithymia at T1 significantly predicted residual change scores of positive and negative emotion regulation difficulties and psychological distress.

#### Emotion Regulation Difficulties as a Mechanism Explaining the Link Between Alexithymia and Psychological Distress

To examine our second hypothesis that increasing difficulties in emotion regulation is a mechanism through which alexithymia increases psychological distress, parallel mediation models were tested using the PROCESS macro for R (model 4; Hayes, 2018). A parallel mediation model allows greater complexity in this context by assessing whether emotion regulation difficulties for either negative or positive emotions provides unique contributions to mediating this relationship. Accordingly, there will be two indirect effects through negative emotion regulation and positive emotion regulation. The difference between these two indirect effects can be tested to investigate whether difficulties in regulating one emotional valence explains more of the relationship between alexithymia and psychological distress than the other. To assess the significance of the indirect effects, 95th percentile bootstrap confidence intervals (CIs) based on 10,000 bootstrap samples were used.

#### Assumptions

The assumptions of multiple regression were tested and addressed appropriately. The assumption of multi-collinearity was met for all analyses, suggesting that all predictor variables (i.e., alexithymia and valence-specific emotion regulation) were meaningfully distinguishable variables in our analysis. The Durbin-Watson test statistic (*p* < 0.01) was used to assess the assumption of independence of residuals. Studentised residuals (with Bonferroni adjustment) were used to identify outliers influencing the regression models. The Breusch-Pagan test (*p* < 0.05) was used to assess the assumption of homoscedasticity. Lastly, the Shapiro–Wilk test (*p* < 0.05) was used to assess the assumption of normality of residuals. Studentised residual outliers were removed. Heteroscedasticity consistent standard errors (HC3) were used if the assumption of homoscedasticity was violated (as recommended by Hayes & Cai, 2007). Bootstrapped confidence intervals were used when the assumption of normality of residuals was violated. R studio [version 4.3.0] was used to conduct all analyses.

## Results

Table 1 presents descriptive statistics and reliability coefficients for all questionnaires at T1 and T2. There was no significant *overall* change in the variables between the two time points. All measures showed excellent Alpha and Omega internal consistency reliabilities (see Supplementary Materials Table S1 for internal consistency reliabilities of the PAQ subscales).

**Table 1:** Descriptive statistics and Cronbach’s alpha and McDonald’s omega reliability coefficients for the administered measures in T1 and T2

	Time 1				Time 2				Difference
Variable	*M*	*SD*	*α*	*ω*	*M*	*SD*	*α*	*ω*	
Total Alexithymia	74.55	27.91	.93	.95	76.57	27.68	.93	.95	*t* = 1.14, *p* = .254
Psychological Distress	13.08	12.38	.94	.95	14.27	12.95	.94	.95	*t* = −1.18, *p* = .240
Negative Emotion Regulation	58.86	18.90	.89	.90	57.28	18.39	.88	.90	*t* = 0.13, *p* = .895
Positive Emotion Regulation	37.42	18.15	.93	.93	37.59	16.50	.91	.91	*t* = 1.55, *p* = .122

No skewness or kurtosis values exceeded ± 2. Differences were conducted using paired-samples *t*-tests (df = 163)

### Alexithymia’s Influence on Change in Emotion Regulation Difficulties and Psychological Distress

The bivariate regressions (Table 2) indicated that alexithymia at T1 provided large effects (see Fey et al., 2023) in predicting future difficulties with regulating positive and negative emotions, and psychological distress. In addition to predicting future levels, individuals with higher levels of alexithymia at T1 exhibited future increases in difficulties regulating negative and positive emotions (medium effects).^1^ Multiple regression analyses and commonality analyses were conducted using the PAQ subscales to examine whether these subscales uniquely predicted the outcomes (Supplementary Materials, Table S2). Overall, all the subscales related to the outcomes, although some nuance was identified; in terms of significant unique contributions to the prediction of all outcomes, only difficulties identifying positive feelings had a significant unique contribution. The shared variance between the subscales (i.e., general alexithymia) explained most of the relationship with the outcomes, rather than the subscales alone (consistent with the status of alexithymia as a coherent multidimensional construct with a strong general factor; Preece & Sikka, 2024). Accordingly, we used the total alexithymia score in the subsequent analysis. Overall, not only does alexithymia relate to emotion regulation difficulties and distress, but alexithymia predicts increased difficulties in regulating emotions.

**Table 2:** The standardised beta-weights of alexithymia at T1 predicting valence-specific emotion regulation and psychological distress at T2 and their changes from T1

	N-ER	P-ER	Psychological Distress
At T2	0.52*** [0.39, 0.66]	0.52*** [0.36, 0.68]†	0.46*** [0.31, 0.61]
Change	0.24** [0.09, 0.39]	0.19* [0.01, 0.37]†	0.09 [−0.06, 0.23]†

N-ER = negative emotion regulation difficulties; P-ER = positive emotion regulation difficulties. Bootstrapped 95% CIs are provided inside square brackets

*p < .05; **p < .01; ***p < .001

†Removed one to three outliers due to significant studentised residuals (with Bonferroni adjustment)

### Emotion Regulation Difficulties as a Mechanism Explaining the Link Between Alexithymia and Psychological Distress

The parallel mediation model had alexithymia scores at T1 entered as the predictor variable, the change scores of negative emotion regulation difficulties and positive emotion regulation difficulties as mediators, and the change score of psychological distress as the outcome variable.

**Fig. 1:**
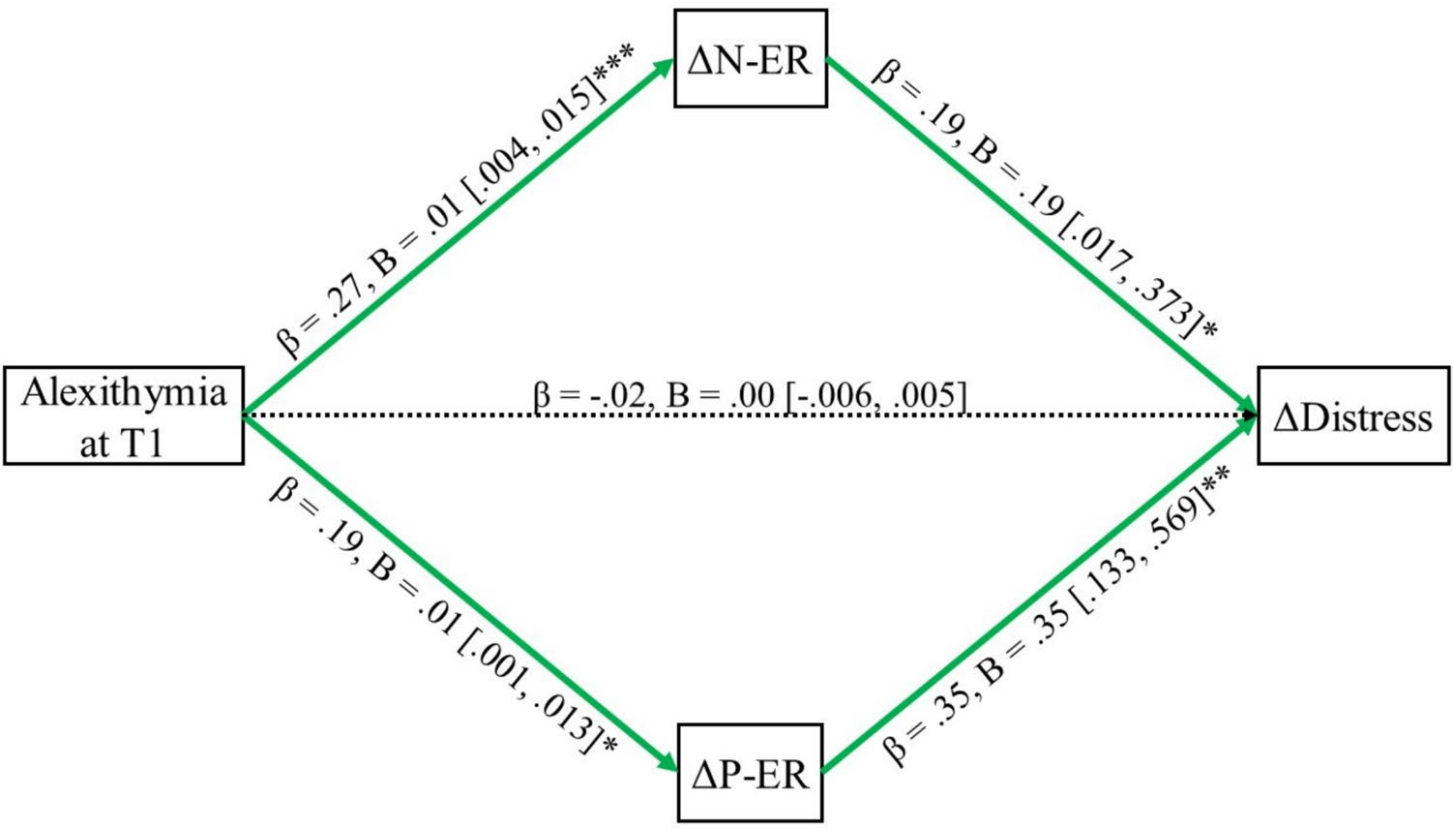
The parallel mediation model of alexithymia at T1 predicting change in psychological distress over time, mediated by change in valence-specific emotion regulation. *Note*: N-ER = Negatively valenced emotion regulation, higher scores indicate worse regulation; P-ER = Positively valenced emotion regulation, higher scores indicate worse regulation. Solid lines indicate significant effects (green = positive), and dotted lines indicate non-significant effects. *p < .05, **p < .001, ***p < .001

The parallel mediation analysis results are shown in Fig. 1.^2^ The model accounted for 18.78% of the variance in psychological distress change, *R*^2^ = .19, F(1, 159) = 4.34, *p* = .039. Individuals’ levels of alexithymia at T1 predicted increased difficulties in regulating positive and negative emotions from T1 to T2, and these increased difficulties in regulating positive and negative emotions, in turn, predicted greater psychological distress from T1 to T2. While alexithymia at T1 did not directly predict changes in psychological distress, indirect effects indicated that alexithymia at T1 indirectly predicted individuals’ increased future psychological distress via increased difficulties in emotion regulation (total indirect effect: *B* = 0.004, *β* = 0.123, 95% CI = [0.037, 0.210]). Changes in the ability to regulate both positive and negative emotions mediated the relationship between alexithymia and changes in psychological distress (positive emotion regulation: *B* = 0.002, *β* = 0.054, 95% CI = [0.004, 0.118]; negative emotion regulation: *B* = 0.002, *β* = 0.068, 95% CI = [0.006, 0.148]). Furthermore, these two indirect effects did not differ significantly from each other, suggesting that both valence domains were important to similar extents (*B* = −0.001, *β* = −0.014, 95% CI = [−0.119, 0.079]). However, the relationship of alexithymia predicting changes in emotion regulation for positive emotions was not robust once controlling for age (*β* = 0.139, *B* = 0.005, 95% CI = [−0.002, 0.013]), leading to alexithymia no longer indirectly increasing psychological distress through change in difficulties regulating positive emotions (*B* = 0.001, *β* = 0.054, 95% CI = [−0.02, 0.14]). There was still no statistical difference between the indirect effect through either positive or negative emotion regulation (*B* = 0.000, *b* = −0.007, 95% CI = [−0.106, 0.078]). Overall, baseline alexithymia levels in Iranian adolescents predicted increased future psychological distress due to increased difficulty in regulating negative emotions and potentially positive emotions.

## Discussion

The present study investigated whether adolescents’ baseline levels of alexithymia predicted future changes in psychological distress and whether this link could be explained by increasing emotion regulation difficulties. Overall, in line with our expectations, we found that alexithymia not only related to emotion regulation difficulties and psychological distress but significantly predicted increased difficulties in regulating both positive and negative emotions. This provides evidence that alexithymia may precede increases in emotion regulation difficulties. Furthermore, the mediation analysis indicated that baseline alexithymia indirectly increased psychological distress via these increased difficulties in emotion regulation.

### How Alexithymia and Psychological Distress are Linked Over Time

While adolescence has been suggested as a pivotal period for emotion regulation development (Blakemore & Mills, 2014), empirical work in this important area has been scarce. Much work focuses on what emotion regulation strategies are used (see Young et al., 2019) rather than their competency in using these strategies effectively. For instance, while suppression is often viewed as a maladaptive strategy, there are contexts in which it is effective (Braet et al., 2024; Khatibi et al., 2021). Accordingly, effective emotion regulation (i.e., good emotion regulation competency) reflects an individual’s ability to successfully utilize a range of strategies, fitting them appropriately to one’s context (Gross, 2015). Our results extend previous research by being the first longitudinal study investigating the relationships between alexithymia and emotion regulation competency. In this context, our novel findings provide important evidence that alexithymia not only relates to adolescents’ current emotion regulation abilities but, importantly, predicts a future increase in emotion regulation difficulties. While studies using cross-sectional data (e.g., Laloyaux et al., 2015; Pollatos & Gramann, 2012; Preece et al., 2022; Swart et al., 2009) highlight that alexithymia and emotion regulation are related, the present results provide evidence to suggest that alexithymia is likely a driving factor in increasing emotion dysregulation for negative emotions, and non-robust indications for also potentially increasing dysregulation in positive emotions. Accordingly, alexithymia may hinder the development of emotion regulation during adolescence, which is likely to have significant cascading effects, such as poorer educational attainment (Martin & Ochsner, 2017), social difficulties (Zeman et al., 2006), and mental health concerns (Schäfer et al., 2017; Young et al., 2019).

Indeed, of these outcomes, our study focused on mental health symptoms through psychological distress (i.e., depression, anxiety, and stress symptoms). Here, a critical question addressed in the current study was about the mechanisms through which alexithymia may be a risk factor for future psychological distress. The attention-appraisal model of alexithymia (Preece & Gross, 2023) and the process model of emotion regulation (Gross, 2015) predict that a key pathway explaining the link between alexithymia and psychological distress should be the impairing impact of alexithymia on emotion regulation. In support of these conceptual perspectives, our mediation analysis revealed that alexithymia appears to indirectly increase psychological distress due to its influence on increasing difficulties regulating negative emotions, and potentially positive emotions. While regulating positive emotions did not robustly mediate this relationship, the influence of regulating either positive or negative emotions mediated the relationship similarly (at least not significantly differently from each other), highlighting the importance of considering both valence domains in this area. This finding is consistent with those of past cross-sectional studies in adults (e.g., Preece et al., 2022) and, importantly, extends this focus on mental health outcomes to adolescents using a more robust longitudinal design.

### Clinical Implications

Past work in the alexithymia field has primarily focused on adults, possibly due to the relative scarcity of validated or reliable alexithymia assessment tools for adolescents throughout much of the construct’s history (e.g., Parker et al., 2010). With the PAQ now enabling robust assessments of alexithymia in adolescents (Trimble et al., 2024), our study extends prior work by focusing on the detrimental effects of alexithymia on mental health during adolescence.

With respect to clinical applications, our findings, therefore, highlight the importance of considering alexithymia in the understanding, assessment, and treatment of mental health issues during this crucial developmental stage. Research has suggested that psychiatric interventions are less effective for individuals with elevated levels of alexithymia (Pinna et al., 2020). However, going further, if alexithymia is neglected, our results suggest that this may contribute significantly to future declines in emotional health. Given that alexithymia manifests as a dimensional construct with individuals having varying levels of alexithymia, as opposed to a categorical construct that is present or absent, many individuals, even those with minimal levels of alexithymia, may benefit from alexithymia-specific interventions, reducing their likelihood of future emotion dysregulation and worse emotional health. Regarding such interventions, the attention-appraisal model emphasises the utility of approaches that increase knowledge of emotions and decrease avoidance of emotions, as these are both fundamental mechanisms contributing to alexithymia (for a discussion of treatment techniques, see Preece & Sikka, 2024). Indeed, there is evidence supporting the effectiveness of such a focus in adults (e.g., Edwards et al., 2018; Norman et al., 2019), and future empirical work will be essential to determine the best ways to target alexithymia in adolescents.

### Strengths and Limitations

The current paper has significant strengths, particularly given its usage of longitudinal data to investigate changes in relevant emotional constructs over time, although limitations should be noted. The sample consisted of Iranian adolescents, and future research will be needed to ascertain whether the current results can be generalised to other cultures (e.g., Chan et al., 2023). While the study design provides some evidence of causality due to investigating alexithymia, emotion regulation, and psychological distress across time, these variables were not directly manipulated. As such, future research that provides alexithymia and emotion regulation specific interventions to adolescents will be able to provide stronger causal inferences. The current results reflect the emotional processing of the general population. Further research will be needed to determine whether the present findings can be generalized to clinical populations or whether the impact of emotional processing differs in clinical samples (e.g., amplified effects). We focused here on depression, anxiety, and stress symptoms, but given the transdiagnostic status of alexithymia, conceptually, these relationships should also be relevant for a wide range of psychopathologies characterised by emotion dysregulation (Mehta et al., 2024). Future research should examine whether the relationships we observed also hold in adolescents for other symptom categories, such as eating disorders, post-traumatic stress disorder, and emerging personality disorders (De Panfilis et al., 2015; Luminet et al., 2021). The study used self-report measures to assess all the constructs of interest. While well-validated, self-report tools have limitations, including memory biases and potential to be impacted if people have low levels of introspective accuracy (Zimmerman, 2024). Future research could investigate whether improving alexithymia through interventions leads to behavioral changes in emotion regulation, as assessed through behavioral or laboratory-based measurements, to examine converging evidence for the relationship between alexithymia and emotion regulation difficulties (Mauss et al., 2005). Lastly, some bodies of research (e.g., Dejonckheere et al., 2019) investigate emotional processes controlling for current affect to examine whether these processes (e.g., alexithymia and emotion regulation) increase mental health symptoms regardless of individuals’ emotional states. Future research could investigate alexithymia’s impact on mental health, controlling for current affect, to isolate the specific dynamics or processes that occur.

## Conclusion

In summary, the current paper provides new insights into the impact of alexithymia on the development of emotion regulation and psychological distress in adolescents. The present results highlight that alexithymia is not merely related to future emotion dysregulation and psychological distress but predicts their increases. An adolescent with high levels of alexithymia is likely to currently be experiencing difficulties regulating their positive and negative emotions, which can lead to greater psychological distress. However, the current findings suggest that, without intervention for their alexithymia, their emotional difficulties and psychological distress may worsen in the future. These findings expand our understanding that alexithymia may hinder the development of emotion regulation in adolescents and that alexithymia may be an essential early intervention target for adolescents experiencing mental health symptoms.

## Supplementary Information

Below is the link to the electronic supplementary material.

Supplementary file1 (DOC 27 KB)

## Footnotes

1 Alexithymia’s influence on depression, anxiety, and stress can be found in the supplementary materials (Table S3).

2 There were violations of the assumptions of homoscedasticity and normally distributed residuals, as such heteroscedasticity consistent standard errors (HC3; as recommended by Hayes & Cai, 2007), bootstrapping, and removal of three outliers (i.e., significant studentized residuals with Bonferroni Adjustment) were used to ensure the current results were not biased (see Knief & Forstmeier, 2021).

## Additional Information

### Data Availability

The current study's de-identified data, data dictionary, and R scripts are openly available in the Open Science Framework at https://osf.io/jwhna/.
