# Supplementary material for: How Alexithymia Increases Mental Health Symptoms in Adolescence: Longitudinal Evidence for the Mediating Role of Emotion Regulation
Source: Affect Sci. 2025 Jul 8;6(3):428–37. doi: 10.1007/s42761-025-00318-9 (PMC12579613; doi:10.1007/s42761-025-00318-9)
Supplement: Supplementary file 1 — Supplementary file1 (DOC 27 KB) [file 42761_2025_318_MOESM1_ESM.doc]

| **Table S1**  *Descriptive statistics and Cronbach’s alpha and McDonald’s omega reliability coefficients for the PAQ subscales at T1 and T2* | | | | | | | | | |
| --- | --- | --- | --- | --- | --- | --- | --- | --- | --- |
|  | Time 1 | | | | Time 2 | | | | |
|  | M | SD | α | ω |  | M | SD | α | ω |
| N-DIF | 13.19 | 5.75 | .75 | .77 |  | 13.65 | 5.69 | .73 | .76 |
| P-DIF | 10.90 | 5.92 | .81 | .82 |  | 10.66 | 5.04 | .72 | .73 |
| N-DDF | 14.54 | 6.33 | .85 | .85 |  | 15.37 | 6.86 | .87 | .87 |
| P-DDF | 12.34 | 5.82 | .79 | .79 |  | 12.66 | 6.38 | .84 | .84 |
| G-EOT | 23.59 | 8.85 | .78 | .79 |  | 24.22 | 8.73 | .79 | .80 |
| *Note.* No skewness or kurtosis values exceeded ±2. N- = negatively valenced; P- = positively valenced; DIF = Difficulties Identifying Feelings; DDF = Difficulties Describing Feelings; G-EOT = General Externally Oriented Thinking; ER = Emotion Regulation. | | | | | | | | | |

**Table S2**

*The total and unique effects (standardised beta-weights) of alexithymia’s components at T1 predicting change in valence-specific emotion regulation and distress.*

| Subscale | T2 N-ER (R^2^ = .38) | | | ΔN-ER (R^2^ = .08) | | |  | T2 P-ER† (R^2^ = .58) | | | ΔP-ER† (R^2^ = .04) | | |  | T2 Distress† (R^2^ = .41) | | | ΔDistress† (R^2^ = .03) | | |
| --- | --- | --- | --- | --- | --- | --- | --- | --- | --- | --- | --- | --- | --- | --- | --- | --- | --- | --- | --- | --- |
|  | Unique | Common | *β* | Unique | Common | *β* |  | Unique | Common | *β* | Unique | Common | *β* |  | Unique | Common | *β* | Unique | Common | *β* |
| N-DIF | .00 | .04 | .31** | .00 | .04 | -.02 |  | .00 | .34 | .11 | .00 | .01 | .06 |  | .01 | .27 | .18 | .00 | .00 | -.07 |
| P-DIF | .00 | .06 | .24* | .00 | .05 | .11 |  | .10 | .46 | .60*** | .00 | .01 | -.08 |  | .05 | .30 | .40*** | .00 | .00 | .02 |
| N-DDF | .00 | .05 | .19 | .00 | .05 | .08 |  | .00 | .28 | -.06 | .00 | .01 | -.04 |  | .00 | .28 | .12 | .00 | .01 | .11 |
| P-DDF | .01 | .06 | .05 | .01 | .06 | .18 |  | .01 | .38 | .13 | .02 | .01 | .26 |  | .01 | .28 | .16 | .01 | .01 | .14 |
| G-EOT | .00 | .02 | -.14 | .00 | .02 | -.08 |  | .00 | .34 | .04 | .00 | .01 | -.02 |  | .01 | .14 | -.18 | .01 | -.01 | -.11 |
| %R^2^ | 20.10% | 79.89% | - | 26.24% | 73.76% | - |  | 19.79% | 80.21% | - | 65.33% | 34.67% | - |  | 17.62% | 82.38% | - | 71.80% | 28.20% | - |

*Note:* N- = negatively valenced; P- = positively valenced; DIF = Difficulties Identifying Feelings; DDF = Difficulties Describing Feelings; G-EOT = General Externally Oriented Thinking; ER = Emotion Regulation. %R^2^ represents the proportion of explained variance that is accounted for by either the subscales uniquely or the common variance in the subscales.

**p* < .05; ***p* < .01; ****p* < .001

†Violated at least one assumption: Removed outliers due to significant studentised residuals (with Bonferroni adjustment; 1-3 outliers removed); normally distributed residuals; heteroscedasticity.

**Table S3**

*The standardised beta-weights of alexithymia at T1 predicting depression, anxiety, and stress at T2 and their changes from T1.*

|  | Depression |  | Anxiety |  | Stress |
| --- | --- | --- | --- | --- | --- |
| At T2 | .46*** [.31, .62] |  | .34*** [.19, .49]† |  | .44*** [.28, .59] |
| Change | .11 [-.05, .27] |  | .05 [-.10, .20]† |  | .09 [-.07, .24] |

*Note:* N-ER = negative emotion regulation; P-ER = positive emotion regulation. Bootstrapped 95% CIs are provided inside square brackets.

**p* < .05; ***p* < .01; ****p* < .001.

†Removed three outliers due to significant studentised residuals (with Bonferroni adjustment).
